# Multi-omics integration identifies Acsm2/ACSM2A deficiency as a metabolic driver of ZBP1-mediated PANoptosis in acute kidney injury

**DOI:** 10.3389/fcell.2026.1936504

**Published:** 2026-09-23

**Authors:** Lijuan Lu, Yuping Qin

**Affiliations:** Department of Nephrology, Nantong Rici Hospital Affiliated to Yangzhou University, Nantong, China

**Keywords:** Acsm2/ACSM2A, acute kidney injury, machine learning, multi-omics integration, PANoptosis

## Abstract

Acute kidney injury (AKI) converges on tubular epithelial cell (TEC) death and metabolic collapse, fueling the transition to chronic kidney disease (CKD), yet the molecular link between metabolic failure and inflammatory cell death remains poorly defined. Here, we integrated cross-model transcriptomics of three mechanistically distinct murine AKI models with single-cell RNA-seq, high-dimensional weighted gene co-expression network analysis, and machine learning feature selection. This multi-omics framework identified acyl-CoA synthetase medium-chain family member 2 (Acsm2), a rate-limiting mitochondrial enzyme for medium-chain fatty acid β-oxidation, as the top conserved hub gene suppressed in injured proximal tubule cells. Acsm2/ACSM2A downregulation was confirmed in mouse AKI models and in human HK-2 cells subjected to oxygen–glucose deprivation/reperfusion (OGD/R). ACSM2A deficiency alone was sufficient to simultaneously activate apoptosis, pyroptosis, and necroptosis, indicative of PANoptosis, whereas restoration of ACSM2A expression attenuated OGD/R-induced cell death. Mechanistically, *ACSM2A* loss provoked mitochondrial oxidative stress and drove the accumulation of Z-form nucleic acids. Scavenging mitochondrial reactive oxygen species or silencing ZBP1 rescued cells from *ACSM2A* knockdown-induced cell death, implicating ZBP1 as a downstream mediator. Collectively, our findings establish *ACSM2A* loss as a trigger of ZBP1-dependent PANoptosis in TECs and position the ACSM2A–ZBP1 axis as a candidate pathogenic mechanism that warrants further investigation as a potential therapeutic node for intercepting the AKI-to-CKD transition.

## Introduction

Acute kidney injury (AKI) is a heterogeneous syndrome that arises from diverse insults, including ischemia-reperfusion, nephrotoxin exposure, and systemic metabolic disturbances (Levey and James, 2017). Despite this etiological diversity, AKI uniformly converges on a common pathological endpoint: tubular epithelial cell (TEC) injury and death, which disrupts renal function and serves as a principal driver of maladaptive repair, renal fibrosis, and the irreversible progression to chronic kidney disease (CKD) (Kellum et al., 2021). Understanding the shared molecular mechanisms that orchestrate TEC fate following disparate renal insults is therefore critical for identifying therapeutic targets capable of intercepting the AKI-to-CKD transition.

Although apoptosis, pyroptosis, necroptosis, and ferroptosis have all been implicated in TEC injury, the cell death landscape in AKI is now appreciated to extend beyond any single modality and their interrelationship and a unified upstream regulatory switch have remained elusive (Sanz et al., 2023). Emerging evidence identifies PANoptosis—a coordinated cell death mechanism that integrates the molecular machinery of apoptosis, pyroptosis, and necroptosis—as a prominent inflammatory death pathway in various diseases (Pandeya and Kanneganti, 2024). The innate immune sensor Z-DNA binding protein 1 (ZBP1) has been recognized as a master upstream regulator of PANoptosis: upon sensing stress-induced Z-conformation nucleic acids, ZBP1 nucleates the assembly of the PANoptosome complex (Liao et al., 2026; Song et al., 2025). However, whether the ZBP1-mediated PANoptosis program operates in renal tubular cells during AKI remains unknown.

A hallmark of AKI is the profound metabolic reprogramming of tubular epithelial cells (Zhu et al., 2022). Healthy proximal tubule cells rely heavily on mitochondrial fatty acid β-oxidation (FAO) and oxidative phosphorylation to meet their extraordinary energy demands, but during injury this metabolic framework collapses, leading to intracellular lipid accumulation, mitochondrial dysfunction, and excessive production of reactive oxygen species (ROS) (Li et al., 2021). While defective FAO has been implicated in renal fibrosis, the specific molecular nodes that link proximal tubule-specific metabolic failure to the initiation of tubular cell death remain incompletely defined. Among the enzymes that gate mitochondrial FAO, Acsm2 (acyl-CoA synthetase medium-chain family member 2) catalyzes the obligate activation of C8–C12 medium-chain fatty acids in the mitochondrial matrix, a rate-limiting step for their entry into β-oxidation (Houten and Wanders, 2010; Watanabe et al., 2020). Notably, the expression and functional significance of Acsm2 in the context of AKI have not been explored.

To address these gaps, we designed an integrated computational and experimental strategy to identify conserved drivers of TEC dysfunction across multiple AKI etiologies and to delineate their downstream death execution mechanisms. In this study, we first performed cross-model transcriptomic analysis of three mechanistically distinct murine AKI models—ischemia-reperfusion injury, cisplatin nephrotoxicity, and folic acid nephropathy—and resolved cell-type-specific contributions using single-cell transcriptomics and high-dimensional gene co-expression network analysis. This multi-omics framework, combined with machine learning feature selection, converged on Acsm2/ACSM2A as the conserved hub gene suppressed in injured proximal tubule cells. Mechanistically, we demonstrate that ACSM2A deficiency triggers mitochondrial oxidative stress, promotes the accumulation of Z-form nucleic acids, and consequently activates ZBP1-dependent PANoptosis in TECs. Our findings establish ACSM2A loss as a central metabolic trigger of inflammatory tubular cell death and position the ACSM2A–ZBP1 axis as a potential candidate axis for preventing AKI-driven CKD progression, pending further *in vivo* validation.

## Results

### Cross-model transcriptomic landscape of acute kidney injury

Acute kidney injury (AKI) encompasses diverse etiological pathways—such as ischemia-reperfusion injury (IRI), nephrotoxicity (e.g., cisplatin), and metabolic stress (e.g., folic acid)—yet these converge on common molecular mechanisms that drive tubular epithelial cell (TEC) dysfunction and death, ultimately predisposing to the progression toward chronic kidney disease (CKD) (Chen et al., 2024; Kellum et al., 2021). To identify cross-model conserved molecular signatures, we performed differential expression analysis on bulk RNA-seq datasets from three established AKI models: GSE153625 (IRI vs. Control, *n* = 4 vs. *n* = 8; Cisplatin vs. Control, *n* = 4 vs. *n* = 8) and GSE65267 (FA vs. Normal, *n* = 15 vs. *n* = 3) (Craciun et al., 2016; Kim et al., 2020). Using DESeq2 with stringent filtering (total counts ≥10 across all samples, FDR <0.05, |log2FC| > 0.5), we identified substantial transcriptional alterations across all three models (Figure 1A). Cisplatin-induced AKI showed 2,593 upregulated and 2,801 downregulated genes; FA nephropathy exhibited 2,195 upregulated and 2,290 downregulated genes; and IRI displayed 2,487 upregulated and 2,128 downregulated genes (Figure 1B).

**FIGURE 1:**
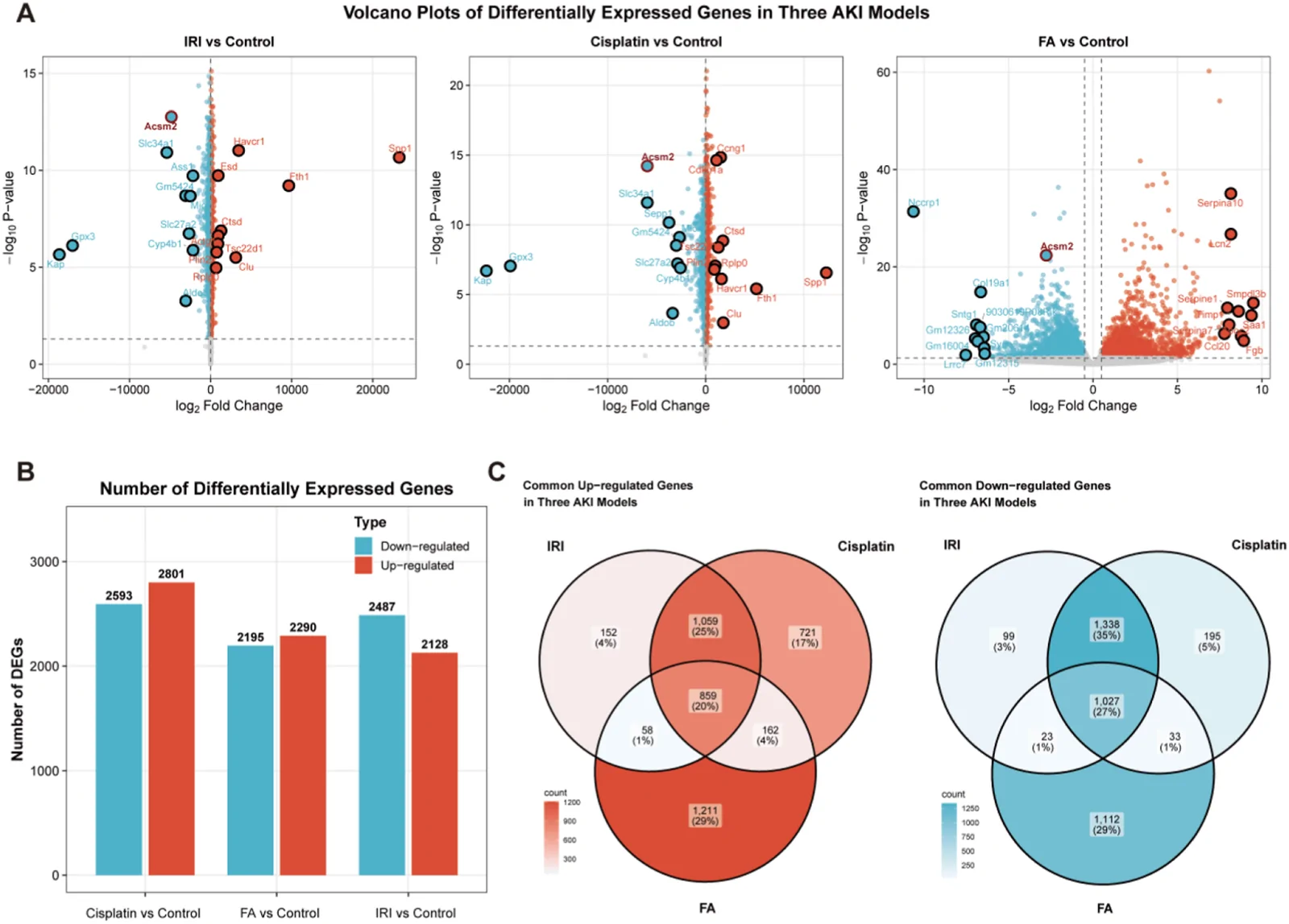
Cross-model transcriptomic analysis identifies conserved dysregulated genes in AKI. **(A)** Volcano plots of differential gene expression for IRI vs. Control (*n* = 4 vs. *n* = 8), Cisplatin vs. Control (*n* = 4 vs. *n* = 8), and FA vs. Normal (*n* = 15 vs. *n* = 3). Red/blue dots: upregulated/downregulated genes (log2FC > 0.5 or < −0.5, FDR <0.05). Top 10 significant genes per direction labeled. **(B)** Bar chart of DEG counts: Cisplatin (2,593 up, 2,801 down), FA (2,195 up, 2,290 down), IRI (2,487 up, 2,128 down). Criteria: FDR <0.05, |log2FC| > 0.5. **(C)** Venn diagrams of shared DEGs: 859 genes (20%) common to all three upregulated sets; 1,027 genes (27%) common to all three downregulated sets.

Intersection analysis revealed a core set of consistently dysregulated genes: 859 genes (20% of total DEGs) were shared among all three upregulated gene sets, and 1,027 genes (27% of total DEGs) were common to all three downregulated gene sets (Figure 1C). This convergent gene signature indicated fundamental molecular pathways underlying AKI regardless of initiating insult.

Gene set enrichment analysis (GSEA) of model-specific DEGs revealed parallel pathway alterations across the three AKI models. In IRI, upregulated genes activated neutrophil chemotaxis (NES = 1.94, padj = 0.0078), granulocyte chemotaxis (NES = 1.94, padj = 0.0078), and Toll-like receptor signaling (NES = 2.02, padj = 0.0013), while downregulated genes suppressed hydrogen peroxide metabolic processes (NES = −1.47, padj = 0.0024), organic acid catabolic processes (NES = −1.58, padj = 0.021), and thyroid hormone synthesis (NES = −1.58, padj = 0.021) (Figure 2A). Cisplatin-induced AKI similarly activated neutrophil migration (NES = 2.01, padj = 0.033), granulocyte migration (NES = 1.99, padj = 0.038), and ECM-receptor interaction (NES = 2.04, padj = 0.0088), with concurrent suppression of hydrogen peroxide metabolism (NES = −1.58, padj = 0.014), organic acid catabolic processes (NES = −1.70, padj = 0.011), and glutathione metabolism (NES = −1.66, padj = 0.027) (Figure 2B). FA nephropathy uniquely activated chromosome separation (NES = 2.15, padj = 2.36 × 10^−14^), TNF signaling (NES = 2.40, padj = 2.25 × 10^−10^), and NF-κB signaling (NES = 2.31, padj = 1.73 × 10^−8^), while suppressing aerobic electron transport chain (NES = −2.45, padj = 5.70 × 10^−8^), amino acid catabolic processes (NES = −2.43, padj = 4.10 × 10^−9^), and oxidative phosphorylation (NES = −2.39, padj = 3.22 × 10^−10^) (Figure 2C).

**FIGURE 2:**
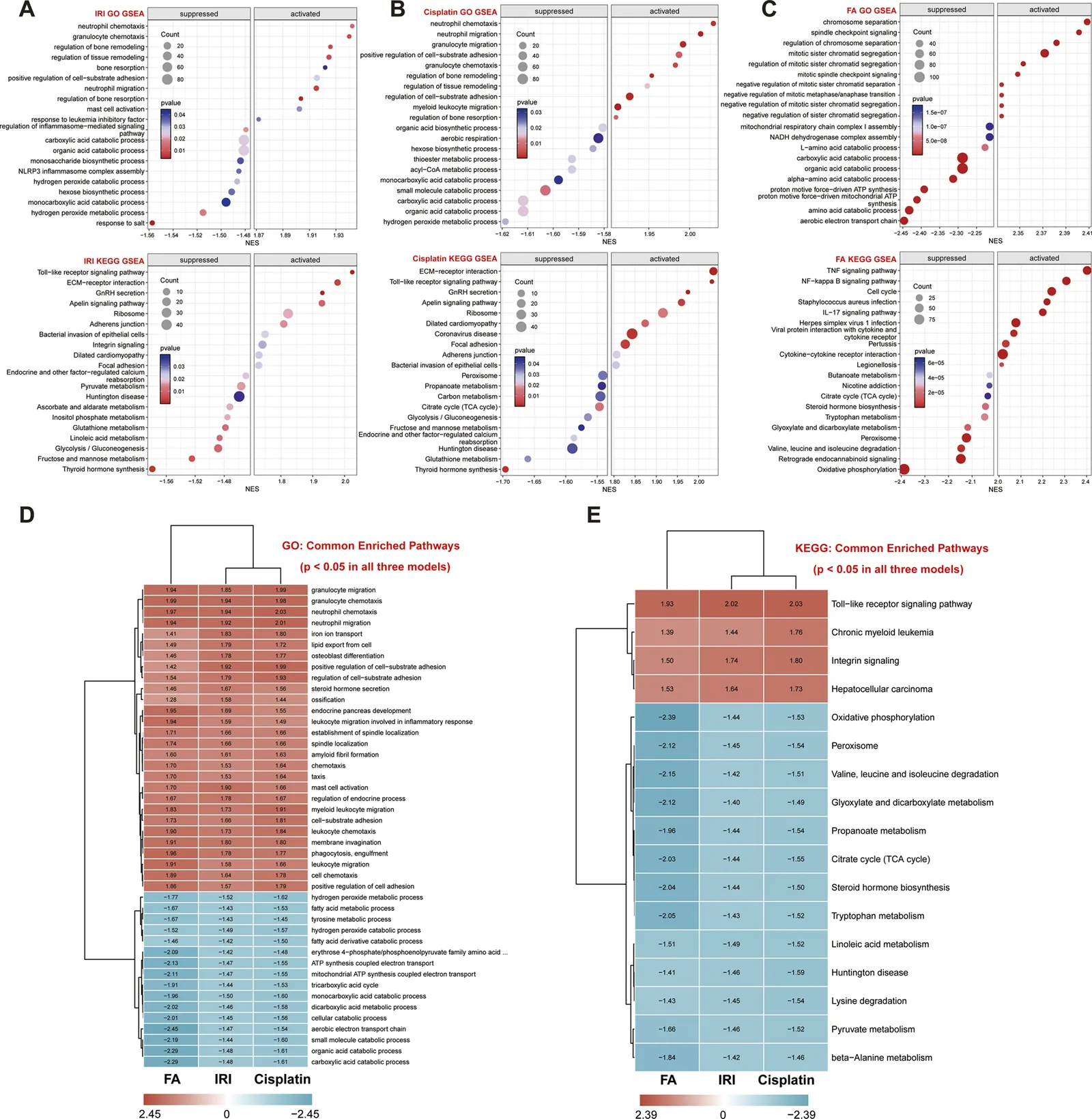
GSEA reveals metabolic-to-inflammatory transition across AKI models. **(A–C)** GSEA dot plots for IRI, Cisplatin, and FA models (GO BP and KEGG pathways). Top 3 enriched pathways per direction shown with NES and padj values. Key findings: neutrophil/granulocyte chemotaxis activated (IRI: NES = 1.94, padj = 0.0078; Cisplatin: NES = 2.01, padj = 0.033; FA: TNF signaling NES = 2.40, padj = 2.25 × 10^−10^); metabolic pathways suppressed (oxidative phosphorylation, TCA cycle, fatty acid metabolism). Dot size: gene set size; color: NES intensity. **(D)** Heatmap of shared GO pathways across models: 28 activated (granulocyte migration, neutrophil chemotaxis, iron ion transport, *etc.*) and 16 suppressed (hydrogen peroxide metabolism, fatty acid metabolism, TCA cycle, *etc.*,; all padj <0.05). Color: red (high NES) to blue (low NES). **(E)** Heatmap of shared KEGG pathways: 4 activated (Toll-like receptor signaling, chronic myeloid leukemia, integrin signaling, hepatocellular carcinoma) and 13 suppressed (oxidative phosphorylation, TCA cycle, peroxisome, valine/leucine/isoleucine degradation, *etc.*,; all padj <0.05).

Cross-model pathway intersection identified 28 GO terms and 4 KEGG pathways that were consistently activated (p < 0.05), including granulocyte migration, neutrophil chemotaxis, iron ion transport, and Toll-like receptor signaling (Figure 2D). Conversely, 16 GO terms and 13 KEGG pathways were uniformly suppressed, encompassing hydrogen peroxide metabolism, fatty acid metabolism, citrate cycle (TCA cycle), oxidative phosphorylation, valine/leucine/isoleucine degradation, and peroxisome function (Figure 2E). This metabolic-to-inflammatory transition signature—characterized by loss of fatty acid oxidation, TCA cycle activity, and mitochondrial respiration coupled with activation of immune cell recruitment and stress signaling—pointed to a fundamental shift in tubular cell homeostasis, positioning metabolic dysfunction as a central driver of AKI pathology.

### Single-cell hdWGCNA reveals PT-specific metabolic and injury modules

To resolve the cellular heterogeneity underlying this transcriptomic signature and identify cell-type-specific modules, we analyzed single-cell RNA-seq data from mouse AKI kidneys (GSE139506) (Rudman-Melnick et al., 2020). Following quality control (genes expressed in >500 cells, cells with >3 genes, mitochondrial gene percentage <25%, ribosomal gene percentage >3%, erythrocyte gene percentage <1%) and Harmony batch correction, unsupervised clustering at resolution 0.5 identified 16 distinct cell populations (Figure 3A). Canonical marker-based annotation classified these populations as endothelial cells (Endo, Pecam1^+^/Kdr^+^/Flt1^+^), cycling cells (Cycling, Mki67^+^/Top2a^+^), podocytes (Podo, Nphs1^+^/Nphs2^+^/Wt1^+^), fibroblasts (Fibro, Pdgfrb^+^/Des^+^), macrophages (Macro, Cd68^+^/C1qa^+^), proximal tubule cells (PT, Slc34a1^+^/Lrp2^+^/Slc27a2^+^), injured proximal tubule (PT-inj, Havcr1^+^/Spp1^+^), inflamed proximal tubule (PT-inflamed, Ccl2^+^/Il6^+^), thick ascending limb (TAL, Slc12a1^+^/Umod^+^/Egf^+^), intercalated cells (CD-IC, Atp6vld^+^), principal cells (CD-PC, Aqp2^+^/Aqp3^+^), smooth muscle cells (SMC, Acta2^+^), and T cells (T, Cd3d^+^/Cd3e^+^) (Figures 3B,C).

**FIGURE 3:**
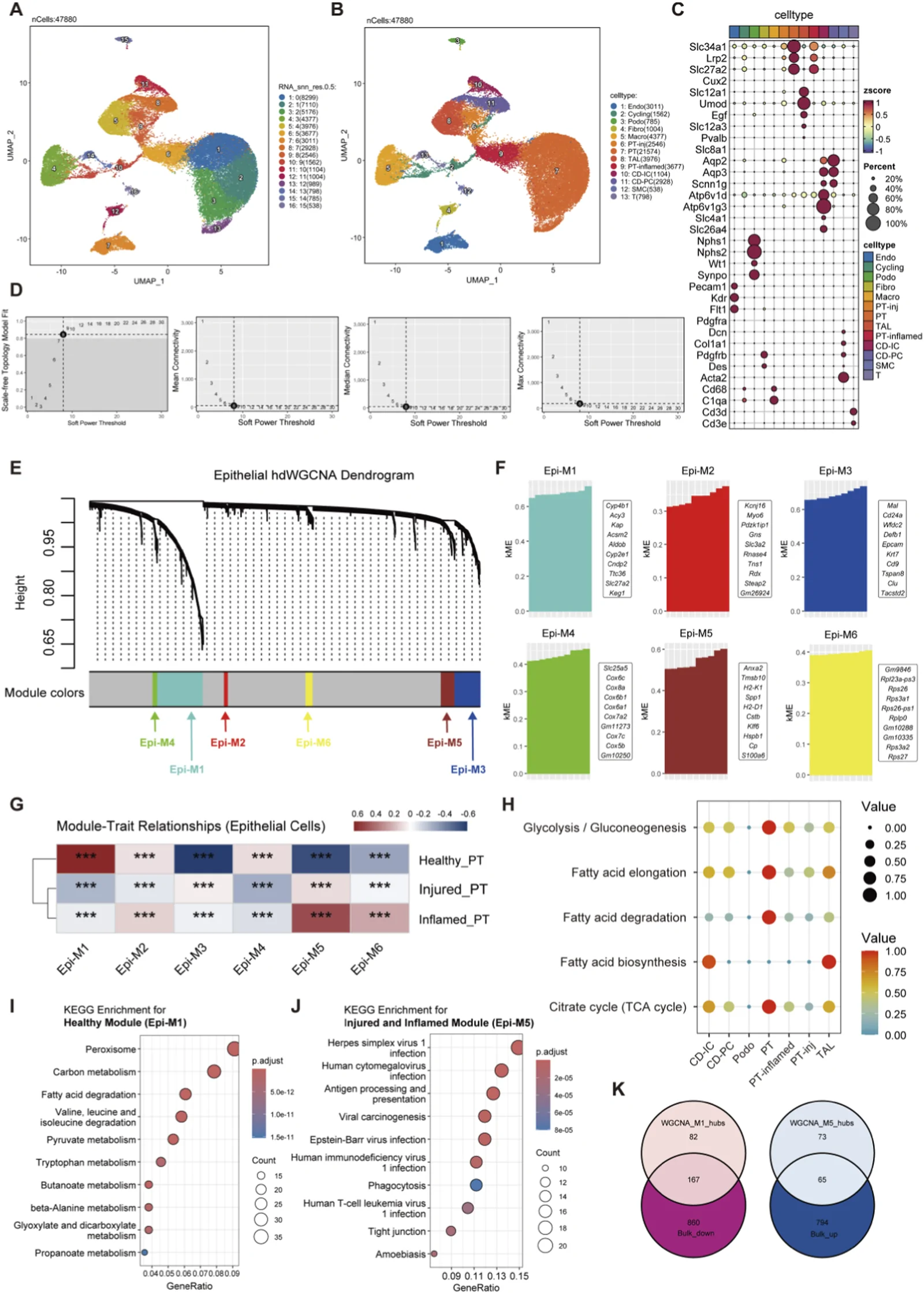
Single-cell hdWGCNA reveals PT-specific metabolic (Epi-M1) and injury (Epi-M5) modules. **(A)** UMAP of 16 cell clusters from GSE139506 (resolution = 0.5). QC filters: genes >500, mitochondria <25%, ribosome >3%. **(B)** UMAP of 12 annotated cell types: Endo, Cycling, Podo, Fibro, Macro, PT, PT-inj, PT-inflamed, TAL, CD-IC, CD-PC, SMC, T. Annotation based on COSG markers. **(C)** Dot plot of canonical markers: Endo (Pecam1, Kdr), Podo (Nphs1, Nphs2), PT (Slc34a1, Lrp2), PT-inj (Havcr1, Spp1), PT-inflamed (Ccl2, Il6), TAL (Slc12a1, Umod), CD-PC (Aqp2). Dot size: expression percentage; color: mean expression. **(D)** Soft threshold power selection (power = 8; scale-free topology fit *R* ^2^ > 0.8). Four panels: *R* ^2^, mean/median/max connectivity vs. power. **(E)** Gene clustering dendrogram with 6 modules (Epi-M1–M6). Parameters: deepSplit = 2, minModuleSize = 30. **(F)** Top 10 hub genes by kME: Epi-M1 (Cyp4b1, Acy3, Kap, Acsm2, Aldob, Cyp2e1, Cndp2, Ttc36, Slc27a2, Keg1); Epi-M5 (Anxa2, Tmsb10, H2-K1, Spp1, H2-D1, Cstb, Klf6, Hspb1, Cp, S100a6). kME threshold >0.3. **(G)** Module-phenotype correlation heatmap (Epi-M1: PT r = 0.72, PT-inj r = −0.68, PT-inflamed r = −0.71; Epi-M5: PT r = −0.65, PT-inj r = 0.69, PT-inflamed r = 0.74; all p < 0.001). Color: red (positive) to blue (negative). **(H)** ScMetabolism pathway scores in PT/PT-inj/PT-inflamed (glycolysis, TCA cycle, fatty acid biosynthesis/degradation/elongation). PT shows higher metabolic activity than injury states (*p < 0.05, p < 0.01, *p < 0.001). **(I,J)** KEGG enrichment: Epi-M1 (propanoate, glyoxylate/dicarboxylate, fatty acid degradation, TCA-related metabolism); Epi-M5 (amoebiasis, tight junction, viral infection, antigen processing). **(K)** Venn diagrams: Epi-M1 hubs ∩ bulk downregulated = 167 genes; Epi-M5 hubs ∩ bulk upregulated = 65 genes; total 232 candidates for ML.

High-dimensional weighted gene co-expression network analysis (hdWGCNA) applied to epithelial cell populations (PT, PT-inj, PT-inflamed, TAL, CD-IC, CD-PC) identified six gene modules (Epi-M1 through Epi-M6) following soft threshold power selection (power = 8, scale-free topology model fit *R* ^2^ > 0.8) (Figures 3D,E). Module-phenotype correlation analysis revealed two modules with distinct cellular associations: Epi-M1 (228 genes) correlated positively with healthy PT (r = 0.72, p < 0.001) and negatively with PT-inj (r = −0.68, p < 0.001) and PT-inflamed (r = −0.71, p < 0.001) (Figure 3G). Whereas Epi-M5 (185 genes) showed inverse correlation, associating with PT-inj (r = 0.69, p < 0.001) and PT-inflamed (r = 0.74, p < 0.001) while negatively correlating with healthy PT (r = −0.65, p < 0.001) (Figure 3G). The top 10 hub genes in Epi-M1 included Cyp4b1, Acy3, Kap, Acsm2, Aldob, Cyp2e1, Cndp2, Ttc36, Slc27a2, and Keg1 (Figure 3F), with Acsm2 positioned as the fourth-ranked hub gene by intramodular connectivity.

KEGG pathway enrichment analysis delineated the functional identities of these modules. Epi-M1 genes enriched for propanoate metabolism, glyoxylate and dicarboxylate metabolism, β-alanine metabolism, butanoate metabolism, tryptophan metabolism, pyruvate metabolism, valine/leucine/isoleucine degradation, fatty acid degradation, and carbon metabolism—all metabolic pathways critical for mitochondrial energy production (Figure 3I). Epi-M5 genes enriched for amoebiasis, tight junction, viral infection pathways, phagocytosis, antigen processing and presentation, and inflammatory response pathways (Figure 3J).

Metabolic activity scoring using scMetabolism across epithelial subpopulations validated the module-phenotype associations. Healthy PT cells maintained high activity in glycolysis/gluconeogenesis, citrate cycle (TCA cycle), fatty acid degradation, and fatty acid elongation, whereas PT-inj and PT-inflamed showed marked suppression of these pathways (Figure 3H). Fatty acid biosynthesis activity remained comparable across PT states, suggesting that the injury phenotype primarily reflected loss of catabolic rather than anabolic metabolic capacity.

Triple intersection analysis—Epi-M1 hub genes with bulk DEG downregulated genes (167 genes), and Epi-M5 hub genes with bulk DEG upregulated genes (65 genes)—identified candidate targets for downstream validation (Figure 3K). This convergence of bulk transcriptomics, single-cell heterogeneity, and network topology positioned Acsm2 at the intersection of three independent analytical approaches, implicating it as a central node in the metabolic collapse of injured proximal tubule cells.

### Machine learning identifies Acsm2 as a conserved diagnostic target

To prioritize candidate genes with diagnostic and therapeutic relevance, we compiled a feature pool of 232 genes from the triple intersection (167 metabolic rescue candidates and 65 pathology inhibition candidates) and applied two complementary machine learning approaches: LASSO regression (10-fold cross-validation) and Random Forest classification (1,000 trees).

LASSO regression identified 8 genes with non-zero coefficients: Acsm2 (coefficient = −0.42), Dnajc12, Gas2, Adhfe1, Arl6ip1, Fmo2, Tpm4, and Cstb (Figure 4C). The regularization path showed progressive coefficient convergence as lambda increased, with optimal lambda selection minimizing binomial deviance (Figures 4A,B). Random Forest feature importance ranking based on MeanDecreaseAccuracy and MeanDecreaseGini scores placed Acsm2 among the top candidates (Figure 4D). Intersection of LASSO-selected genes and Random Forest top-10 importance-ranked genes converged on a single gene: Acsm2 (Figure 4E).

**FIGURE 4:**
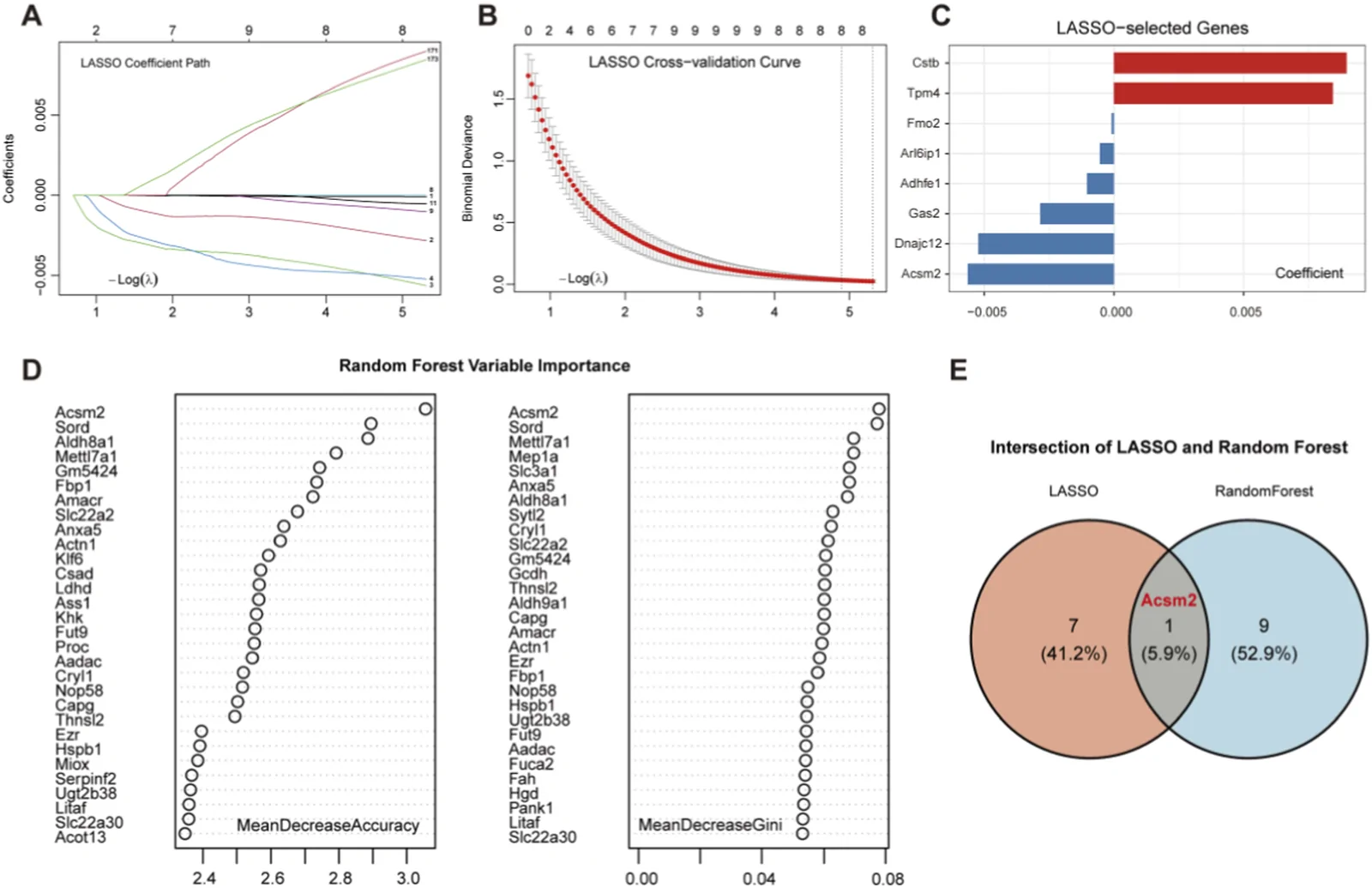
Machine learning converges on Acsm2 as diagnostic target. **(A)** LASSO coefficient path (coefficients vs. regularization). Optimal lambda selected by 10-fold CV (glmnet, binomial family). **(B)** LASSO CV curve (binomial deviance vs. lambda). Lambda. min and lambda.1se shown. **(C)** 8 LASSO-selected genes with non-zero coefficients: Acsm2 (coef = −0.42), Dnajc12, Gas2, Adhfe1, Arl6ip1, Fmo2, Tpm4, Cstb. Bar length: coefficient magnitude. **(D)** Random Forest importance (ntree = 1,000). Bubble plots: MeanDecreaseAccuracy and MeanDecreaseGini for top 30 genes (Acsm2 ranked high). Bubble size/color: importance score. **(E)** Venn diagram: LASSO 8 genes ∩ RF top 10 → Acsm2 (sole convergent target).

This convergence across three orthogonal computational methods—bulk transcriptomic intersection, network hub ranking, and dual machine learning feature selection—established Acsm2 as the primary molecular signature of AKI-induced metabolic dysfunction. The consistent identification through independent approaches, coupled with its positioning as a high-connectivity node in the Epi-M1 metabolic module, motivated focused experimental validation of Acsm2 functional role in tubular epithelial cell injury and its potential as a therapeutic target for preventing AKI-to-CKD progression.

Performance of the model was then quantified and externally validated by receiver-operating-characteristic (ROC) analysis with cross-validation (Supplementary Figure S1). In the training cohort (GSE153625; 8 sham vs. 8 AKI), the LASSO panel achieved an AUC of 1.000 under leave-one-out cross-validation (Supplementary Figure S1A). Applied with all coefficients frozen to the fully independent folic-acid cohort GSE65267 (3 normal vs. 15 AKI), which was not used at any feature-selection step, the LASSO panel achieved an AUC of 0.933 (95% CI 0.812–1.000), and Acsm2 alone achieved an AUC of 0.978 (95% CI 0.916–1.000) with sensitivity 0.93, specificity 1.00, positive predictive value 1.00, negative predictive value 0.75, and accuracy 0.94 at the Youden-index cutoff (Supplementary Figure S1B). Feature selection of Acsm2 was perfectly stable: across 100 random seeds and three cross-validation schemes ({4, 5, 10}-fold; 300 resampling runs in total), Acsm2 entered the LASSO model in 100% of runs, ranking fourth of 232 candidates by selection frequency and eighth by median |coefficient| (Supplementary Figure S1C). Re-derivation of the pipeline with the current implementations (glmnet 5.0) recovered all eight originally selected LASSO genes. Single-gene analysis of all 232 candidates confirmed that Acsm2 itself retained high diagnostic accuracy in both the training and the external cohort (Supplementary Figure S1D; Supplementary Table S1). Consistent with the diagnostic power of multi-omics strategies recently demonstrated in renal ischemia-reperfusion injury (Zhang et al., 2025), these analyses support Acsm2 as a robust diagnostic candidate rather than an artifact of small-sample feature selection.

### Acsm2 is consistently suppressed in AKI tubular epithelial cells

Through cross-transcriptomic analysis of three AKI models—IRI, folic acid, and cisplatin—we identified Acsm2, a key enzyme in mitochondrial fatty acid β-oxidation, as the key gene that is significantly downregulated (Figures 1A–C, 4E).

Quantitatively, Acsm2 suppression was strong and significant in all three models (log2FC = −1.96, P = 3.7 × 10^−4^, IRI; log2FC = −3.50, P = 1.4 × 10^−4^, cisplatin; log2FC = −2.77, FDR = 2.3 × 10^−20^, FA), with the largest effect size in nephrotoxic cisplatin injury. Temporal analysis of the FA time course showed that Acsm2 expression fell from a baseline of 10.50 to 8.21 within 1 day, reached its nadir at day 2 (7.37), and recovered only partially by day 14 (9.71), remaining below baseline throughout the observation window; in the single-cell IRI time course, proximal-tubule pseudo-bulk Acsm2 was lowest at day 7 after injury. In both time courses the PANoptosis program followed the reciprocal trajectory (Supplementary Figure S2D). These data indicate that Acsm2 downregulation is a general, early and sustained response of injured proximal tubule cells across AKI etiologies rather than a model-specific phenomenon.

Systematic correlation analysis further demonstrated that Acsm2 expression is inversely associated with the PANoptosis program at both tissue and single-cell resolution (Supplementary Figure S2). In the cisplatin model, Acsm2 correlated negatively with all seven PANoptosis genes analyzed (Spearman rho = −0.888, P = 9.0 × 10^−5^ for Zbp1; Pearson r = −0.94 to −0.98 for Ripk3, Mlkl, Gsdmd and Casp3); in the IRI model, correlations were negative for Ripk3 (r = −0.91), Mlkl (r = −0.88) and Casp3 (rho = −0.657, P = 0.024); and in the FA model for Ripk3 (rho = −0.475, P = 0.046) and Mlkl (rho = −0.486, P = 0.043) (Supplementary Figure S2A). At single-cell resolution (25,824 proximal tubule and injured proximal tubule cells from GSE139506), Acsm2 was negatively correlated with every PANoptosis effector tested (e.g., Gsdmd rho = −0.193, P = 2.1 × 10^−215^; Zbp1 rho = −0.119, P = 3.1 × 10^−82^), whereas in healthy proximal tubule cells none of the correlations reached significance (Supplementary Figure S2B,C). Pseudo-bulk analysis across the IRI time course confirmed negative correlations for Gsdmd (r = −0.890, P = 0.017), Casp3 (r = −0.861, P = 0.028) and Nlrp3 (r = −0.822, P = 0.045) (Supplementary Figure S2d). These consistent inverse relationships support the proposed antagonism between tubular Acsm2 activity and ZBP1-mediated PANoptosis during AKI progression.

Human relevance was further examined in an independent public cohort of kidney transplant biopsies (GSE30718 (Famulski et al., 2012)) comprising 28 biopsies with acute kidney injury/delayed graft function, 11 pristine protocol biopsies from stable grafts, and 8 normal nephrectomy tissues. ACSM2A/B expression was significantly lower in AKI biopsies than in controls (log2FC = −0.34, P = 0.018, FDR = 0.035) and distinguished AKI from controls with an AUC of 0.707 (95% CI 0.553–0.860) - comparable with the established injury biomarker HAVCR1 (KIM-1) measured in the same samples (AUC 0.701; DeLong P = 0.955). Across all 47 biopsies, ACSM2A/B expression was inversely correlated with five of the seven PANoptosis genes analyzed (GSDMD rho = −0.49, P = 5.1 × 10^−4^; MLKL rho = −0.47, P = 7.9 × 10^−4^; CASP1 rho = −0.43; NLRP3 rho = −0.43; RIPK3 rho = −0.40; all P < 0.01), and these inverse relationships persisted within AKI biopsies alone (GSDMD, NLRP3 and MLKL, all P < 0.05), whereas ZBP1 and CASP3 did not correlate significantly in this cohort. The canonical injury biomarkers HAVCR1 (KIM-1), LCN2, VCAM1 and SPP1 were all significantly induced, validating the cohort (Supplementary Figure S3). These results establish that ACSM2A downregulation and its inverse relationship with PANoptosis executors are detectable in human AKI tissue, supporting the translational relevance of our findings.

To further validate these bioinformatic findings and the critical role of Acsm2 in AKI-induced disease progression, we established the three AKI mouse models described above (Figures 5A,F,K). Compared with control mice, mice treated with IRI, folic acid, or cisplatin showed significantly elevated serum creatinine and blood urea nitrogen (BUN) levels on day 2 after treatment, indicating successful induction of all three AKI models (Figures 5B,C,G,H,L,G). We subsequently isolated TECs from mice on day 2 after AKI induction for analysis. The results showed that Acsm2 expression was significantly decreased at both the RNA and protein levels in all three AKI models relative to the respective controls (Figures 5D,E,I,JN–I). To further corroborate these findings *in vitro*, we employed the human renal tubular epithelial cell line HK-2 and simulated AKI injury using an oxygen–glucose deprivation/reperfusion (OGD/R) model, then examined ACSM2A expression. The results revealed that both RNA and protein levels of ACSM2A were markedly reduced after OGD/R treatment compared with the control group (Figures 5P,Q). Notably, a similar significant decrease in ACSM2A expression was also observed in primary human renal tubular epithelial cells following OGD/R treatment (Figure 5R), further corroborating the consistency of this regulation across different cell models.

**FIGURE 5:**
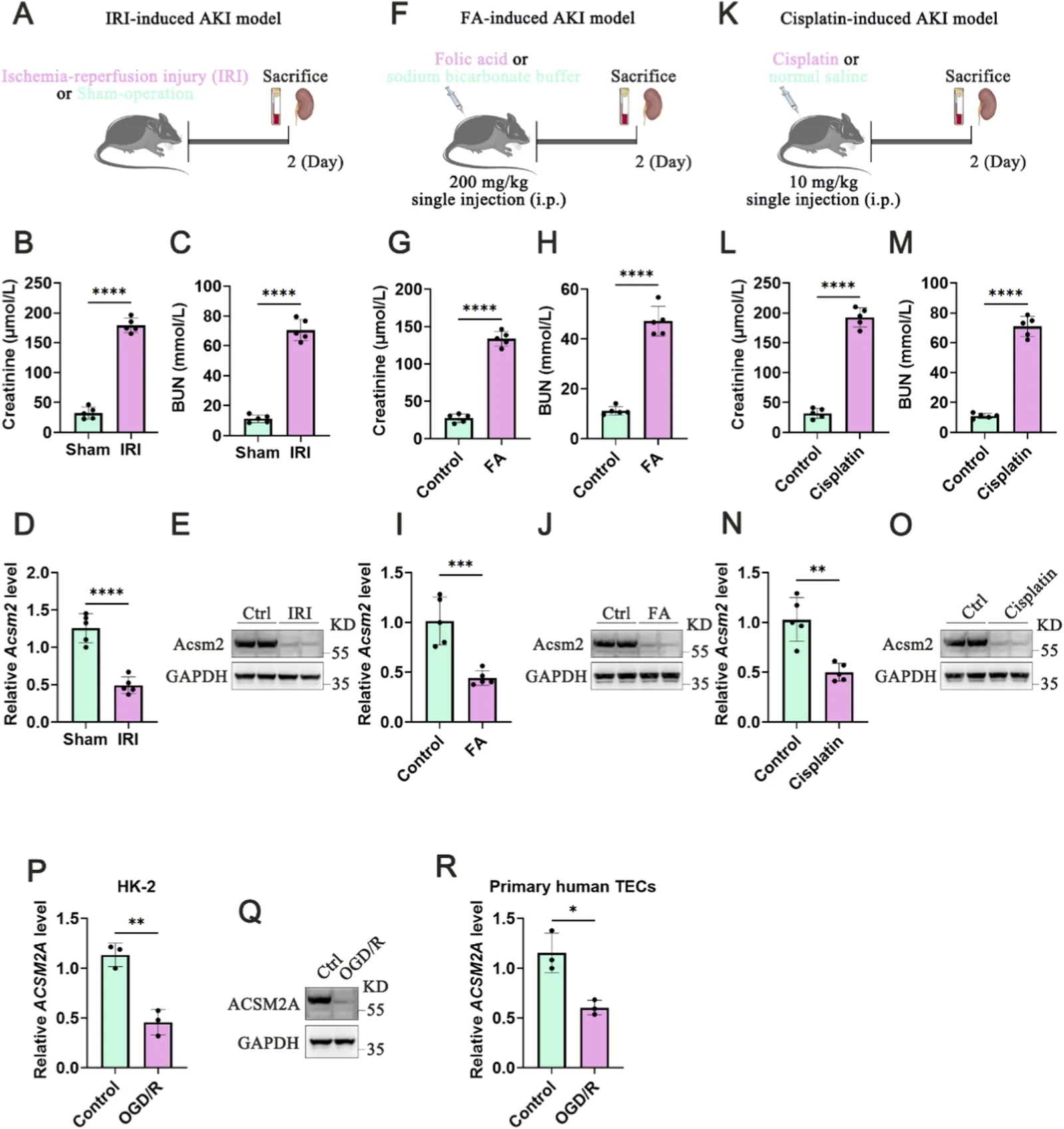
Acsm2/ACSM2A is downregulated in renal tubular epithelial cells from three distinct AKI mouse models and in OGD/R-induced cell models. **(A)** A schematic diagram showing the construction of the IRI-induced AKI mouse model. **(B,C)** Renal function was assessed by measuring serum creatinine **(B)** and BUN **(C)** in a mouse model of IRI-induced AKI (mice *n* = 5). **(D)** Transcriptional level of *Acsm2* in IRI-induced AKI mouse model (mice *n* = 5). **(E)** Western blot analysis of Acsm2 protein expression in a mouse model of IRI-induced AKI. **(F)** A schematic diagram showing the construction of the FA-induced AKI mouse model. **(G,H)** Renal function was assessed by measuring serum creatinine **(G)** and BUN **(H)** in a mouse model of FA-induced AKI (mice *n* = 5). **(I)** Transcriptional level of *Acsm2* in FA-induced AKI mouse model (mice *n* = 5). **(J)** Western blot analysis of Acsm2 protein expression in a mouse model of FA-induced AKI. **(K)** A schematic diagram showing the construction of the Cisplatin-induced AKI mouse model. **(L,M)** Renal function was assessed by measuring serum creatinine **(L)** and BUN **(M)** in a mouse model of Cisplatin-induced AKI (mice *n* = 5). **(N)** Transcriptional level of *Acsm2* in Cisplatin-induced AKI mouse model (mice *n* = 5). **(O)** Western blot analysis of Acsm2 protein expression in a mouse model of Cisplatin-induced AKI. **(P)** Transcriptional level of *ACSM2A* in OGD/R-induced HK-2 cell models (*n* = 3). **(Q)** Western blot analysis of ACSM2A protein expression in OGD/R-induced HK-2 cell models. **(R)** Transcriptional level of *ACSM2A* in OGD/R-induced primary human TECs models (*n* = 3). Data are representative of three independent experiments (E, J, O, Q). Error bars are shown as mean ± SD with *n* = 3 (P, R) or *n* = 5 (B-D, G-I, L-N) biological replicates. Two-tailed unpaired Student’s t-test (B-D, G-I, L-N, P, R). *P < 0.05, **P < 0.01, ***P < 0.001, ****P < 0.0001, NS, not significant.

Taken together, these findings indicate that Acsm2/ACSM2A downregulation is a common and conserved molecular feature of tubular epithelial cells across AKI of different etiologies, and it may play a critical role in the transition from AKI to CKD.

### ACSM2A downregulation mediates OGD/R-induced PANoptosis in tubular epithelial cells

The progression from AKI to CKD is a major cause of end-stage renal failure, and the death of TECs plays a critical role in this process (Li et al., 2022). Using an oxygen–glucose deprivation/reoxygenation (OGD/R) model in human renal tubular epithelial HK-2 cells, we measured lactate dehydrogenase (LDH) release and found that OGD/R treatment significantly increased LDH release compared with the control group (Figure 6A), indicating that OGD/R promotes lytic cell death in HK-2 cells. SYTOX Green staining for total dead cells further confirmed that OGD/R caused significant cell death (Figure 6B).

**FIGURE 6:**
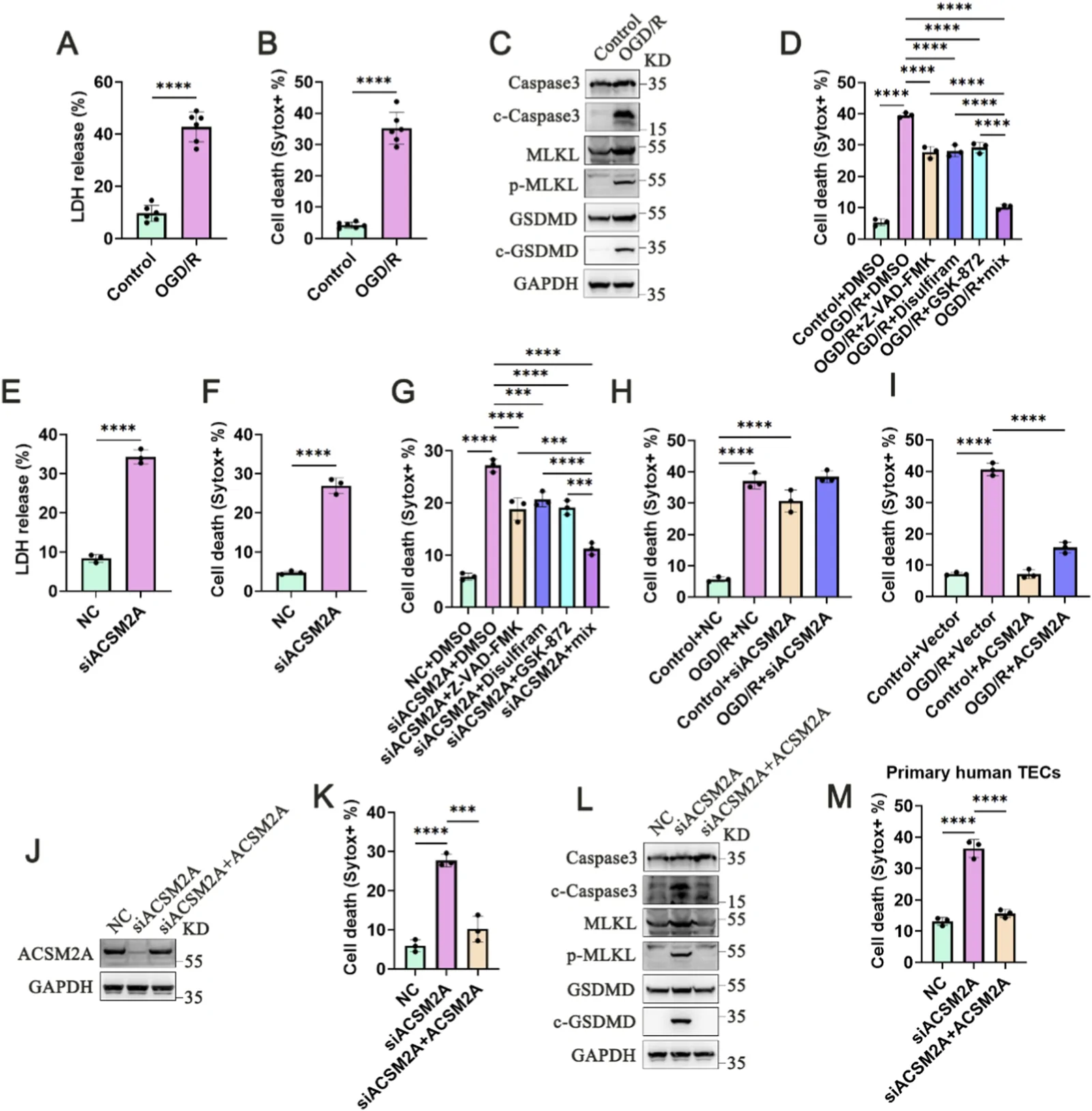
ACSM2A downregulation promotes PANoptosis in renal tubular epithelial cells during AKI. **(A)** LDH release from control and OGD/R-treated HK-2 cells as a measure of cytotoxicity (*n* = 6). **(B)** Cell death quantification in control and OGD/R-treated HK-2 cells (*n* = 6). **(C)** Western blot analysis of Caspase 3, Cleaved-caspase 3 (c-caspase3), MLKL, Phosphorylated-MLKL (p-MLKL), GSDMD and Cleaved-GSDMD (c-GSDMD) protein expression in control and OGD/R-treated HK-2 cells. **(D)** Cell death quantification in control and OGD/R-treated HK-2 cells, pre-treated with dimethylsulfoxide (DMSO; control), 20 µM Z-VAD-FMK, 10 µM Disulfiram, 10 µM GSK-872 or a combination of all three inhibitors for 1 h (*n* = 3). **(E)** Cytotoxicity assessed by LDH release from HK-2 cells following siRNA-mediated knockdown of ACSM2A for 48 h (*n* = 3). **(F)** Cell death quantification in HK-2 cells following siRNA-mediated knockdown of ACSM2A for 48 h (*n* = 3). **(G)** Cell death quantification in HK-2 cells transfected with ACSM2A siRNA for 24 h, followed by treatment with DMSO (control), 20 µM Z-VAD-FMK, 10 µM disulfiram, 10 µM GSK-872, or a combination of all three inhibitors for an additional 24 h (*n* = 3). **(H)** Cell death quantification in HK-2 cells transfected with ACSM2A siRNA for 48 h and subsequently subjected to control or OGD/R treatment (*n* = 3). **(I)** Cell death quantification in HK-2 cells overexpressing of ACSM2A for 48 h and subsequently subjected to control or OGD/R treatment (*n* = 3). **(J)** Western blot analysis of ACSM2A and GAPDH were conducted in control HK-2 cells, cells subjected to ACSM2A knockdown, and cells in which ACSM2A was overexpressed subsequent to ACSM2A knockdown. **(K)** Cell death quantification in HK-2 cells under the indicated treatment conditions, as shown in Fig. **(J)** (*n* = 3). **(L)** Western blot analysis of Caspase 3, Cleaved-caspase 3 (c-caspase3), MLKL, Phosphorylated-MLKL (p-MLKL), GSDMD and Cleaved-GSDMD (c-GSDMD) protein expression in HK-2 cells under the indicated treatment conditions, as shown in Fig. **(J)**. **(M)** Cell death quantification in primary human TECs under the indicated treatment conditions, as shown in Fig. J (*n* = 3). Data are representative of three independent experiments **(C, J, L)**. Error bars are shown as mean ± SD with *n* = 3 **(D-I, K, M)** or *n* = 6 **(A,B)** biological replicates. Two-tailed unpaired Student’s t-test **(A,B,E,F)**. One-way ANOVA with Tukey’s multiple comparisons test **(D, G, H, I, K, M)**. *P < 0.05, **P < 0.01, ***P < 0.001, ****P < 0.0001, NS, not significant.

To define the specific mode of TEC death under AKI conditions, we performed Western blot analysis. The results showed that OGD/R treatment induced proteolytic activation of caspase-3, GSDMD, and MLKL, as evidenced by the appearance of cleaved caspase-3, GSDMD N-terminal fragment, and phosphorylated MLKL (Figure 6C). Notably, pharmacological inhibition of any single pathway only partially reduced OGD/R-induced cell death, whereas combined blockade of all three pathways nearly completely rescued cell death (Figure 6D), indicating that these pathways are functionally coordinated rather than merely coexisting. This interdependent activation pattern is consistent with the operational definition of PANoptosis, although direct demonstration of PANoptosome formation was not performed in this study.

Given our earlier finding that Acsm2/ACSM2A was significantly downregulated in TECs after AKI (Figure 5), we next examined whether OGD/R-induced cell death is mediated by Acsm2/ACSM2A reduction. SiRNA-mediated knockdown of ACSM2A in HK-2 cells led to a pronounced increase in cell death (Figures 6E,F). Similarly, inhibitors of apoptosis, pyroptosis, or necroptosis alone only partially alleviated cell death caused by ACSM2A knockdown, whereas triple inhibition nearly completely abrogated this death (Figure 6G). These findings are consistent with a coordinated, PANoptosis-like cell death program, although direct evidence for PANoptosome assembly is lacking. Notably, even in HK-2 cells not subjected to OGD/R, ACSM2A knockdown alone was sufficient to induce significant cell death (Figure 6H). Conversely, overexpression of ACSM2A in OGD/R-treated HK-2 cells significantly reduced the level of cell death (Figure 6I). Finally, we performed a rescue experiment: restoring ACSM2A expression in ACSM2A-knockdown HK-2 cells (Figure 6J) markedly reversed the PANoptosis caused by ACSM2A deficiency (Figures 6K,L). Similarly, in primary human renal tubular epithelial cells, knockdown of ACSM2A expression also led to increased cell death, whereas restoration of ACSM2A expression effectively reduced the level of cell death (Figure 6M).

In summary, downregulation of Acsm2/ACSM2A is a key event in OGD/R-induced PANoptosis of renal tubular epithelial cells. Acsm2/ACSM2A deficiency is sufficient on its own to trigger mixed cell death, and restoration of its expression significantly attenuates OGD/R-elicited PANoptosis. These rescue experiments support the specificity of the ACSM2A knockdown phenotype, although we acknowledge that the use of a single siRNA and overexpression-based rescue does not completely exclude off-target effects; future studies with independent siRNAs or CRISPR-based approaches are warranted. Taken together, these findings suggest that Acsm2/ACSM2A may represent a candidate target for intervening in the AKI-to-CKD transition.

### ACSM2A loss-induced mitochondrial oxidative stress activates ZBP1-dependent PANoptosis

Acsm2/ACSM2A is a core medium-chain fatty acid-activating enzyme localized in the mitochondrial matrix (Watanabe et al., 2020); however, how its loss of function triggers PANoptosis remains to be elucidated. The reaction catalyzed by Acsm2/ACSM2A constitutes the sole rate-limiting step for the entry of C8–C12 medium-chain fatty acids into mitochondrial β-oxidation (Houten and Wanders, 2010). Measurement of intracellular ATP levels showed that ACSM2A knockdown significantly reduced ATP production compared with control cells, whereas restoration of ACSM2A expression fully rescued ATP levels back to baseline **(** Figure 7A). We hypothesized that Acsm2/ACSM2A deficiency causes medium-chain fatty acid accumulation. The accumulation of unmetabolized C8–C12 fatty acyl intermediates would be expected to damage mitochondrial membranes and respiratory complexes, potentially impairing, ETC., function and inducing electron leak (Agrawal et al., 2017; Alhumaidi et al., 2025; Amaral and Wajner, 2020). The combined lipotoxic injury and electron leak then could drive a burst of mitochondrial ROS production. To test the above hypothesis, we first examined HK-2 cells after ACSM2A silencing using MitoSOX Red, a mitochondrial superoxide-specific probe. The results showed that ACSM2A knockdown markedly increased mitochondrial ROS levels in HK-2 cells, whereas restoration of ACSM2A expression reduced the elevated ROS (Figures 7B,C). Consistently, assessment with DCFH-DA, a total cellular ROS indicator, also revealed that ACSM2A knockdown caused a significant increase in ROS, and re-expression of ACSM2A restored ROS to the pre-treatment level (Figures 7D,E). These data indicate that the mitochondrial oxidative stress storm triggered by ACSM2A loss may be an early upstream event that activates PANoptosis.

**FIGURE 7:**
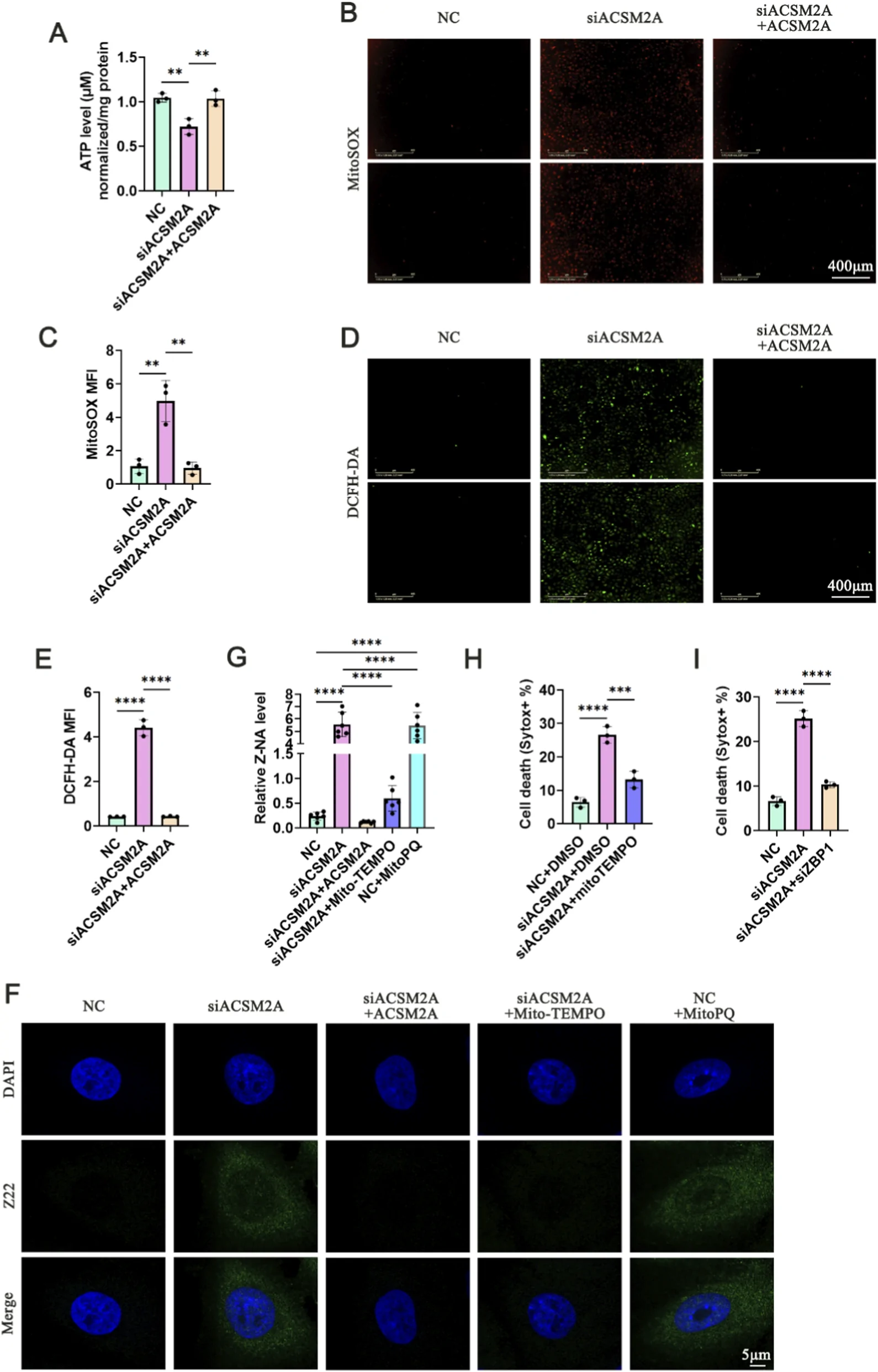
ACSM2A deficiency in renal tubular epithelial cells promotes ZBP1-mediated PANoptosis by inducing mitochondrial oxidative stress and Z-nucleic acid accumulation. **(A)** Intracellular ATP levels in control HK-2 cells, ACSM2A-knockdown cells, and ACSM2A-knockdown cells with ACSM2A overexpression. **(B,C)** Representative fluorescence images **(B)** and quantitative analysis **(C)** of MitoSox in control HK-2 cells, ACSM2A-knockdown cells, and ACSM2A-knockdown cells with ACSM2A overexpression. Red fluorescence indicates MitoSox (mitochondrial superoxide). **(D,E)** Representative fluorescence images **(D)** and quantitative analysis **(E)** of DCFH-DA in control HK-2 cells, ACSM2A-knockdown cells, and ACSM2A-knockdown cells with ACSM2A overexpression. Green fluorescence indicates DCFH-DA (ROS). **(F,G)** Representative fluorescence images **(F)** and quantification **(G)** of Z22 levels in control HK-2 cells, ACSM2A-knockdown cells, ACSM2A-knockdown cells with ACSM2A overexpression, ACSM2A-knockdown cells treated with mito-TEMPO (10 μM) for 24 h and control HK-2 cells treated with MitoPQ (10 μM) for 12 h. Green fluorescence indicates Z22 (Z-NA). Blue fluorescence indicates DAPI (nucleus). **(H)** Cell death quantification in control HK-2 cells, ACSM2A-knockdown cells, and ACSM2A-knockdown cells treated with mito-TEMPO (10 μM) for 24 h (*n* = 3). **(I)** Cell death quantification in control HK-2 cells, ACSM2A-knockdown cells, and ACSM2A-knockdown cells with ZBP1 knockdown (*n* = 3). Data are representative of three independent experiments **(B,D,F)**. Error bars are shown as mean ± SD with *n* = 3 **(A,C,E,H,I)** or *n* = 6 **(G)** biological replicates. One-way ANOVA with Tukey’s multiple comparisons test **(A,C,E,G,H,I)**. *P < 0.05, **P < 0.01, ***P < 0.001, ****P < 0.0001, NS, not significant.

Recent studies have shown that mitochondrial oxidative stress can induce mtDNA breaks and the formation of Z-conformation nucleic acids, which in turn activate ZBP1(Song et al., 2025). ZBP1 (Z-DNA binding protein 1) is a key upstream sensor of PANoptosis and initiates the process by assembling the ZBP1-dependent PANoptosome complex (Liao et al., 2026; Pandeya and Kanneganti, 2024). To further corroborate this hypothesis, we assessed the levels of Z-form nucleic acids in cells by immunofluorescence. To determine whether mitochondrial ROS is sufficient to induce Z-form nucleic acids, we treated control HK-2 cells with MitoPQ, a mitochondria-targeted redox cycler that selectively elevates mitochondrial superoxide. MitoPQ treatment markedly induced a significant accumulation of Z-form nucleic acids, as detected by Z22 immunofluorescence (Figures 7F,G). And compared with the control group, ACSM2A knockdown led to the appearance of pronounced Z-form nucleic acids, particularly in the cytoplasm; in contrast, re-expression of ACSM2A in ACSM2A-knockdown cells or scavenging mitochondrial ROS with the mitochondria-targeted antioxidant mito-TEMPO reduced the formation of Z-form nucleic acids (Figures 7F,G). Consistently, eliminating mitochondria-derived ROS with mito-TEMPO or knocking down ZBP1 expression via siRNA also attenuated cell death induced by ACSM2A knockdown (Figures 7H,I).

In summary, our results demonstrate that Acsm2/ACSM2A deficiency promotes the generation of Z-form nucleic acids by inducing mitochondrial oxidative stress, which subsequently engages ZBP1-dependent PANoptosis, ultimately leading to renal tubular epithelial cell death and driving the progression from AKI to CKD. These data are consistent with ZBP1 functioning downstream of ACSM2A deficiency-induced mitochondrial oxidative stress, although direct biochemical evidence for ZBP1 activation and PANoptosome assembly remains to be established.

## Discussion

The transition from acute kidney injury to chronic kidney disease is driven by a maladaptive interplay between tubular epithelial cell death and metabolic demise, yet the molecular sentinels that couple metabolic failure to inflammatory cell death have remained elusive. By integrating cross-model transcriptomics, single-cell gene co-expression networks, and dual machine learning feature selection, we identified acyl-CoA synthetase medium-chain family member 2 (Acsm2/ACSM2A) as a conserved hub gene suppressed in injured proximal tubule cells. Our study establishes that Acsm2/ACSM2A deficiency operates as a metabolic trigger that connects impaired mitochondrial fatty acid β-oxidation to the activation of ZBP1-dependent PANoptosis—a synchronized inflammatory death program encompassing apoptosis, pyroptosis, and necroptosis. The superior protection conferred by triple inhibition compared to single-pathway blockade, together with the rescue by ZBP1 silencing, supports the conclusion that ACSM2A deficiency engages a coordinated cell death program consistent with PANoptosis rather than parallel activation of independent death modalities. These findings delineate an ACSM2A–ZBP1 signaling axis that is central to tubular cell fate determination and identify a candidate node for therapeutic intervention, although the long-term efficacy and safety of targeting this axis *in vivo* remain to be established.

The convergence of multiple unbiased computational strategies on Acsm2 underscores the power of multi-omics integration in identifying pathogenic drivers that transcend individual AKI etiologies. Despite the distinct initiating mechanisms of ischemia-reperfusion, cisplatin toxicity, and folic acid nephropathy, all three models uniformly suppressed a core metabolic program featuring fatty acid degradation, the TCA cycle, and oxidative phosphorylation. Within this program, hdWGCNA pinpointed an epithelial metabolic module (Epi-M1) whose hub genes—including Acsm2—were selectively extinguished in injured and inflamed proximal tubule cells while being preserved in healthy counterparts. The subsequent selection of Acsm2 as the unique intersecting gene between LASSO regression and Random Forest models reinforces its diagnostic relevance and argues that its loss is not merely an epiphenomenon of injury but a functionally consequential event. Prior studies have implicated defective fatty acid oxidation in renal fibrosis and lipid accumulation (Kang et al., 2015; Miguel et al., 2021), yet the specific molecular nodes through which proximal tubule metabolic collapse feeds into cell death execution remained unspecified. By demonstrating that ACSM2A downregulation alone is sufficient to simultaneously engage apoptotic, pyroptotic, and necroptotic machinery, we fill this gap and establish a direct causal link between a single FAO enzyme deficiency and a multimodal cell death phenotype. This convergence has now been quantitatively validated: Acsm2 was selected by LASSO in 300 of 300 resampling runs, retained high diagnostic accuracy in an independent external AKI cohort (AUC = 0.978), was inversely correlated with ZBP1 and multiple PANoptosis effectors across bulk and single-cell datasets, and was downregulated in human AKI biopsies, where its expression was inversely correlated with multiple PANoptosis effectors (Supplementary Figure 1–3), consistent with the diagnostic power of multi-omics strategies in AKI demonstrated by recent integrative studies (Zhang et al., 2025).

A key question raised by these observations is how the loss of a mitochondrial metabolic enzyme precipitates inflammatory cell death. Our data support a model in which ACSM2A deficiency provokes mitochondrial oxidative stress that, in turn, promotes the accumulation of Z-form nucleic acids and subsequent ZBP1 activation. We propose that Acsm2 deficiency leads to intramitochondrial accumulation of medium-chain fatty acyl species, which may directly damage respiratory complexes and foster electron leak, ultimately driving ROS production (Amaral and Wajner, 2020). The resulting surge in mitochondrial ROS, which we verified using MitoSOX and DCFH-DA probes, creates an environment conducive to oxidative damage of mitochondrial DNA and the generation of Z-conformation nucleic acids. ZBP1 has recently emerged as a master sensor of such Z-nucleic acids (Jiao et al., 2020; Yuan et al., 2022). Our demonstration that scavenging mitochondrial ROS with mito-TEMPO or silencing ZBP1 abolishes ACSM2A loss-driven PANoptosis supports a model in which ZBP1 acts downstream of mitochondrial oxidative damage, identifying a metabolic stress-to-innate immune signaling cascade that has not previously been appreciated in kidney tubular cells. However, direct physical interactions between ZBP1 and other PANoptosome components, such as RIPK3, RIPK1, and caspase-8, were not examined in this study and remain an important area for future investigation.

Notably, a recent study on nephron-sparing surgery-related AKI (NRI) revealed that PP2A-mediated suppression of the ERK–PPAR-α/PGC-1α signaling axis is a central mechanism driving the decline in proximal tubular fatty acid oxidation capacity, lipid accumulation, and mitochondrial dysfunction (Zhang et al., 2026). That work demonstrated that maintaining PPAR-α/PGC-1α transcriptional activity is critical for preserving tubular metabolic homeostasis across various ischemic and non-ischemic AKI settings. Given that PPAR-α is a direct transcriptional regulator of multiple fatty acid oxidation enzymes, including Acsm2/ACSM2A, it is reasonable to propose that the downregulation of Acsm2/ACSM2A observed in our study may represent one of the downstream consequences of PP2A–ERK–PPAR-α pathway inactivation. This cascade of dysregulation—from upstream signaling to downstream metabolic enzymes—may provide a unifying molecular explanation for impaired fatty acid oxidation following AKI of diverse etiologies.

These mechanistic insights hold direct therapeutic implications. The ACSM2A–ZBP1 axis offers multiple potential intervention points: restoring ACSM2A expression or activity, reducing mitochondrial ROS with targeted antioxidants, or blocking Z-nucleic acid accumulation. Our observation that overexpression of ACSM2A attenuates OGD/R-elicited cell death and that re-expression reverses PANoptosis in ACSM2A -deficient cells suggests that therapies aimed at preserving or restoring proximal tubule FAO capacity could limit the initial wave of cell loss that precipitates maladaptive repair and fibrosis. Given the current lack of specific therapies to halt the AKI-to-CKD transition, targeting this metabolic-innate immune crosstalk may represent a promising strategy. Regarding the clinical feasibility of targeting this axis, several considerations should be noted. Enhancing fatty acid oxidation, for example, by restoring ACSM2A activity, may require proximal tubule-restricted delivery strategies to avoid systemic metabolic side effects such as excessive lipid catabolism or off-target effects in other FAO-dependent tissues. Conversely, inhibiting ZBP1 could compromise innate immune responses, particularly antiviral defense, given the established role of ZBP1 in sensing viral Z-nucleic acids. Therefore, any future therapeutic approach based on this axis would likely require transient, kidney-targeted intervention, ideally guided by biomarkers of Acsm2 loss or ZBP1 activation, to maximize benefit while minimizing systemic immune or metabolic risk.

Several limitations of this study should be acknowledged. First, the *in vivo* functional requirement for Acsm2 in tubular PANoptosis and AKI-to-CKD progression remains to be demonstrated using cell-type-specific Acsm2 knockout or knock-in models, which would definitively establish causality. Although our *in vitro* gain- and loss-of-function experiments and cross-model correlative analyses strongly support a causal role, direct *in vivo* manipulation is still needed. Second, although we provide functional evidence of mitochondrial bioenergetic failure—namely increased mitochondrial ROS and reduced ATP levels—direct measurement of medium-chain fatty acid species, acyl-CoAs, and mitochondrial oxygen consumption rate (OCR) was not performed in this study. Such targeted metabolomic and respirometric analyses would provide a more complete picture of FAO impairment and are important future directions. Third, although we used the well-characterized Z22 antibody to detect Z-form nucleic acids, antibody-based detection alone does not distinguish Z-DNA from Z-RNA, and we did not perform orthogonal validation such as nuclease digestion or competition assays. Future studies using Zα domain probes, nuclease sensitivity assays, and quantitative dot blot analyses will be needed to precisely define the nature and relative abundance of Z-DNA and Z-RNA species in this model. Fourth, although our necessity and sufficiency experiments establish that mitochondrial ROS is both required and sufficient for Z-form nucleic acid accumulation downstream of ACSM2A loss, we did not perform high-resolution time-course experiments to define the precise temporal sequence of these events. Future studies using inducible ACSM2A deletion and live-cell imaging of mitochondrial ROS and Z-form nucleic acids will be needed to dissect the kinetics of this signaling axis. Finally, the upstream signals that repress Acsm2/ACSM2A transcription during AKI and the precise lipid species that mediate mitochondrial damage warrant deeper metabolic and epigenomic characterization. We also did not systematically test whether individual inflammatory cytokines or generic tubular injury stimuli are sufficient to suppress Acsm2 expression. These are important future directions that will help distinguish upstream drivers from downstream consequences and identify the specific injury-associated signals that repress Acsm2/ACSM2A.

In conclusion, our study identifies Acsm2/ACSM2A as a critical metabolic sentinel whose loss couples proximal tubule mitochondrial stress to ZBP1-dependent PANoptosis, providing a unified mechanism that bridges the metabolic and inflammatory facets of AKI. Further exploration of the Acsm2/ACSM2A–ZBP1 axis in preclinical models may yield novel diagnostic biomarkers and candidate therapeutic targets, although their clinical potential remains to be validated in long-term *in vivo* studies.

## Methods

### Transcriptomic data acquisition and processing

Bulk RNA-seq datasets were obtained from Gene Expression Omnibus (GEO). GSE153625 provided transcriptomic profiles for ischemia-reperfusion injury (IRI) and cisplatin-induced AKI models in mice, comprising 8 control samples, 4 IRI samples, and 4 cisplatin samples (the rhabdomyolysis arm of the series was not analyzed). GSE65267 contained data for folic acid (FA)-induced nephropathy, with 3 normal control samples and 15 FA-treated samples across a 14-day time course. The two datasets were processed separately according to their data type. For GSE153625, which provides within-series normalized (TPM-like) expression values, differential expression was analyzed on log2-transformed data (log2 (x+1)) using linear models with empirical-Bayes moderated t-statistics (limma); for GSE65267, raw integer counts were normalized by size factors and analyzed with the DESeq2 (version 1.38.3) negative-binomial Wald test, filtering genes with total counts below 10 prior to analysis. Because each AKI model was contrasted only against its own concurrent controls and no sample-level integration across datasets was performed, no cross-dataset batch correction was required; cross-model consistency was assessed at the gene level (intersections of DEG lists and signed log2 fold-changes), which is insensitive to between-platform batch effects. Genes were classified as significantly dysregulated based on false discovery rate (FDR) < 0.05 (Benjamini–Hochberg correction) and absolute log2 fold change >0.5, identified separately for each model, and shared dysregulated genes across all three AKI models were determined by intersection analysis via the ‘intersect ()` function in R, with overlap percentages calculated relative to total DEG counts.

### Gene set enrichment analysis

Gene set enrichment analysis (GSEA) was performed on model-specific differential expression results using the clusterProfiler package (version 4.10.0). Gene lists ranked by log2 fold change were submitted to GSEA with 1,000 permutations. Gene ontology biological process (GO BP) terms and Kyoto Encyclopedia of Genes and Genomes (KEGG) pathways were used as reference databases. Normalized enrichment scores (NES) and adjusted p-values (padj, Benjamini–Hochberg correction) were calculated for each pathway. Pathways with padj <0.05 were considered significantly enriched. Top-ranked pathways (top 10 upregulated and top 10 downregulated for both GO and KEGG) were extracted for each model. Cross-model pathway intersection was performed to identify consistently activated or suppressed pathways across all three AKI models, using pathway term overlap analysis with significance threshold p < 0.05.

### Single-cell RNA-seq analysis and hdWGCNA

Single-cell RNA-seq data from mouse AKI kidney was obtained from GSE139506. Data processing was performed using Seurat (version 5.0.1) in R. Quality control filters were applied: cells were retained if gene count >500, mitochondrial gene percentage <25%, ribosomal gene percentage >3%, and erythrocyte gene percentage <1%. Genes detected in fewer than 3 cells were removed. Count matrices were normalized using ‘NormalizeData ()` with log normalization, and highly variable genes were identified using ‘FindVariableFeatures ()` with selection method “vst”. Data integration across samples was performed using Harmony (version 1.0.0) to correct batch effects, with reduction set to “pca” and group. by.var set to sample origin. Dimensional reduction via principal component analysis (PCA) used the top 30 principal components. Cell clustering was performed using ‘FindNeighbors ()` with dims = 1:30 and ‘FindClusters ()` with resolution = 0.5. Visualization was achieved through uniform manifold approximation and projection (UMAP) using ‘RunUMAP ()` with dims = 1:30.

Cell type annotation was performed using canonical marker genes identified through COSG (version 1.2.0) differential expression analysis between clusters. Marker genes for each cell type were validated against known literature: endothelial cells (Pecam1, Kdr, Flt1), cycling cells (Mki67, Top2a), podocytes (Nphs1, Nphs2, Wt1, Synpo), fibroblasts (Pdgfrb, Des), macrophages (Cd68, C1qa), proximal tubule (PT: Slc34a1, Lrp2, Slc27a2), injured proximal tubule (PT-inj: Havcr1/KIM-1, Spp1), inflamed proximal tubule (PT-inflamed: Ccl2, Il6), thick ascending limb (TAL: Slc12a1, Umod, Egf, Slc12a3), intercalated cells (CD-IC: Atp6vld, Atp6v1g3, Slc4a1, Slc26a4), principal cells (CD-PC: Aqp2, Aqp3, Scnn1g), smooth muscle cells (SMC: Acta2), and T cells (Cd3d, Cd3e).

High-dimensional weighted gene co-expression network analysis (hdWGCNA) was performed using the hdWGCNA package (version 0.1.0) on epithelial cell populations (PT, PT-inj, PT-inflamed, TAL, CD-IC, CD-PC). Metacells were constructed using ‘SetMetacells ()` with k = 20 nearest neighbors, reduction = “pca”, and ident. group = “celltype”. Soft threshold power selection was performed using ‘TestSoftPowers ()` to achieve scale-free topology model fit *R* ^2^ > 0.8, resulting in optimal power = 8. Module detection was executed using ‘FindModules ()` with deepSplit = 2 and minModuleSize = 30. Module eigengenes (MEs) were calculated as principal component 1 of each module’s expression matrix. Module-phenotype correlation analysis was performed by correlating module eigengene scores with cell state annotations (PT, PT-inj, PT-inflamed) using Pearson correlation, with significance assessed via two-sided t-test. Hub genes for each module were identified by ranking intramodular connectivity (kME) values, with top 10 genes extracted per module.

### Metabolic pathway analysis

Metabolic activity scoring across epithelial subpopulations was performed using scMetabolism (version 1.0) with default parameters. Gene sets for glycolysis/gluconeogenesis, citrate cycle (TCA cycle), fatty acid biosynthesis, fatty acid degradation, and fatty acid elongation were obtained from KEGG metabolic pathway annotations. Metabolism scores were calculated as average expression of pathway-specific gene sets scaled by Z-score normalization across all cells. Differences in metabolic activity between PT, PT-inj, and PT-inflamed populations were assessed using Wilcoxon rank-sum test with Benjamini–Hochberg correction for multiple comparisons.

### Machine learning feature selection

Candidate gene pools were compiled from triple intersection analysis: (1) bulk DEG shared downregulated genes intersected with Epi-M1 hub genes (kME >0.3) yielded 167 metabolic rescue candidates; (2) bulk DEG shared upregulated genes intersected with Epi-M5 hub genes (kME >0.3) yielded 65 pathology inhibition candidates, totaling 232 candidate genes. Feature selection was performed using two complementary approaches. LASSO (Least Absolute Shrinkage and Selection Operator) regression was implemented using glmnet (version 4.1.8) with binomial family, 10-fold cross-validation (nfolds = 10), and lambda selected at minimum cross-validated deviance. Genes with non-zero coefficients at optimal lambda were retained. Random Forest classification was performed using randomForest (version 4.7.1.1) with ntree = 1,000, importance = TRUE, and proximity = TRUE. Feature importance was assessed using MeanDecreaseAccuracy and MeanDecreaseGini metrics, with top 10 genes ranked by importance. Final target gene selection required presence in both LASSO non-zero coefficient set and Random Forest top-10 importance list, identified via intersection analysis.

### Statistical analysis for bioinformatics

All statistical analyses were performed in R (version 4.3.1). Differential expression significance was assessed using DESeq2’s Wald test with Benjamini–Hochberg false discovery rate correction. GSEA significance thresholds required padj <0.05. Module-phenotype correlations were calculated using Pearson correlation with two-sided significance testing. Multiple comparison corrections applied Benjamini–Hochberg procedure where appropriate. Data visualization was performed using ggplot2 (version 3.4.4), patchwork (version 1.1.3) for multi-panel layouts, and GseaVis for GSEA dot plots. All computational analyses were executed on a workstation with 128 GB RAM and Intel Core i7 processor.

### Correlation analysis between Acsm2 and PANoptosis-related genes

Spearman and Pearson correlations between Acsm2 and seven PANoptosis-related genes (Zbp1, Ripk3, Mlkl, Gsdmd, Casp1, Casp3, Nlrp3) were computed in each bulk RNA-seq model on log2-transformed data (GSE153625: log2 (x+1); GSE65267: log2(CPM+1)) and, at single-cell resolution, across proximal tubule cells (PT and injured PT clusters) of GSE139506 using log-normalized expression, with injured-PT-only and per-time-point pseudo-bulk analyses as sensitivity checks. P values are two-sided and reported exactly.

### Machine-learning model evaluation and external validation

LASSO and Random Forest models were trained on GSE153625 (8 sham vs. 8 AKI, IRI and cisplatin pooled) as described above. Performance was assessed by ROC analysis (pROC): apparent and leave-one-out cross-validated AUC in the training cohort; external validation in the independent FA cohort GSE65267 (3 normal vs. 15 AKI) with LASSO coefficients frozen at training values (linear predictor from cv. glmnet at lambda. min); and single-gene ROC analysis of all 232 candidates, with each gene’s diagnostic orientation fixed in the training cohort before external evaluation. Sensitivity, specificity, positive and negative predictive values were computed at the Youden-index optimal cutoff. Selection robustness was quantified over 100 random seeds x {4, 5, 10}-fold cross-validation (300 runs; selection frequency and |coefficient| rank), and Random Forest robustness over 20 seeds × {500, 1000, 2000} trees. Model evaluation and robustness analyses were performed in R 4.5.3 with glmnet 5.0, randomForest 4.7–1.2 and pROC 1.18.5.

### Human AKI dataset validation

ACSM2A dysregulation in human AKI was assessed in GSE30718 (Famulski et al., 2012), a public cohort of 47 kidney biopsies profiled on the Affymetrix HG-U133 Plus 2.0 array (RMA-normalized log2 expression), comprising 28 biopsies with acute kidney injury/delayed graft function, 11 pristine protocol biopsies from stable grafts, and 8 normal nephrectomy tissues. Probesets were mapped to genes using Ensembl BioMart cross-referenced with the hgu133pLus2. db annotation; where several probesets mapped to one gene, the probeset with the highest mean expression was used. Because ACSM2A and ACSM2B are highly similar paralogs that share most probesets on this platform, results are reported as ACSM2A/B (primary probeset 230554_at, the highest-expressed of the three shared probesets), with the ACSM2B-specific probeset 241915_at analyzed in parallel. AKI-versus-control differences were tested with Wilcoxon rank-sum and Welch t tests, and correlations with PANoptosis genes (ZBP1, RIPK3, MLKL, GSDMD, CASP1, CASP3, NLRP3) with Spearman correlation. HAVCR1 (KIM-1), LCN2, VCAM1 and SPP1 were analyzed as positive-control injury biomarkers to validate the cohort.

### Experimental animals

Animal experiments were performed in compliance with the guidelines and protocols approved by the Animal Ethics Committee of Nantong Rici Hospital Affiliated to Yangzhou University. All procedures were in accordance with the Guidelines for the Care and Use of Laboratory Animals. The animals were maintained under specific pathogen-free conditions with controlled temperature (25 ± 1 °C) and humidity (55 ± 5%), subjected to standardized 12 h/12 h light-dark photoperiod cycles. Food and water were provided *ad libitum* throughout the experimental period.

### Renal ischemia-reperfusion injury model establishment

Male C57BL/6 mice (8–12 weeks old) were anesthetized by intraperitoneal injection of sodium pentobarbital (40 mg/kg; Sigma-Aldrich, P3761). After confirming the absence of the pedal reflex, the mice were placed on a heating pad to maintain body temperature at 37 °C. Through a midline incision, right unilateral nephrectomy was performed, and acute kidney injury (AKI) was induced by clamping the left renal pedicle with a non-traumatic aneurysm clip for 30 min. The ischemic duration was based on previous ischemia-reperfusion injury (IRI) studies in mice (Zheng T. et al., 2021; Zheng Z. et al., 2021). Sham-operated mice underwent the same surgical procedure, including right nephrectomy and exposure of the left renal artery, but without clamping. At 48 h after reperfusion, the mice were euthanized by an overdose of sodium pentobarbital followed by cervical dislocation, and kidney and blood samples were collected for further analysis.

### The FA-induced or cisplatin-induced AKI model establishment

To establish the folic acid (FA)- or cisplatin-induced acute kidney injury (AKI) model, mice received a single intraperitoneal injection of FA (200 mg/kg; MCE, HY-16637) or cisplatin (10 mg/kg; MCE, HY-17394). FA was dissolved in sodium bicarbonate buffer, and cisplatin was dissolved in normal saline. Control mice received an equivalent volume of the corresponding vehicle (sodium bicarbonate buffer or normal saline). At 2 days after injection, the mice were sacrificed to assess AKI, and blood and kidney samples were collected.

### Assessment of renal function

Blood samples were collected and centrifuged (3000rpm, 10 min, 4 °C) to obtain serum. Creatinine (Cr) and blood urea nitrogen (BUN) concentrations were quantified using spectrophotometric assay kits (C011-2–1/C013-2–1; Nanjing Jiancheng Bioengineering Institute, China) according to manufacturer’s protocols. Absorbance measurements were performed at 510 nm (Cr) and 640 nm (BUN) using a microplate reader with internal calibration standards.

### Primary renal TECs were isolated from mice

Primary renal tubular epithelial cells (TECs) were isolated from mice as previously described (Li et al., 2025; Xing et al., 2026). Briefly, excised renal cortex tissue was incubated with collagenase type I (4 mg/mL; Sigma-Aldrich, SCR103) at 37 °C for 30 min. After centrifugation, the cells were resuspended in PBS and sequentially filtered through 70-μm and 40-μm cell strainers. Primary TECs were cultured in RPMI-1640 medium supplemented with 10% fetal bovine serum and 1% penicillin–streptomycin at 37 °C in a 5% CO_2_ incubator.

### Cell culture and treatment

HK-2 human proximal tubular epithelial cells (Procell, Wuhan, China; Cat. No. CL-0109) and primary human TECs (Procell, Wuhan, China; Cat. No. CP-H193) were cultured in Dulbecco’s modified Eagle’s medium/nutrient mixture F-12 (DMEM/F-12; Gibco, United States of America) supplemented with 10% fetal bovine serum (Gibco), 100 U/mL penicillin, and 100 μg/mL streptomycin (Gibco). All cells were maintained at 37 °C in a humidified atmosphere of 5% CO_2_.

To mimic ischemia/reperfusion injury *in vitro*, an oxygen–glucose deprivation/reoxygenation (OGD/R) model was used. HK-2 cells and primary human TECs were incubated in glucose-free DMEM (Gibco) and placed in a hypoxia chamber (STEMCELL Technologies, Vancouver, BC, Canada) flushed with an anoxic gas mixture (95% N_2_, 5% CO_2_) at 37 °C for 12 h. Thereafter, the medium was replaced with normal complete medium, and the cells were transferred to a normoxic incubator (95% air, 5% CO_2_) for 2 h of reoxygenation.

The following reagents were added as described: 20 µM Z-VAD-FMK (HY-16658B; MedChemExpress), 10 µM disulfiram (HY-B0240; MedChemExpress), 10 µM GSK-872 (HY-101872; MedChemExpress), 10 µM mito-TEMPO (HY-112879; MedChemExpress), 10 µM MitoPQ (HY-130278; MedChemExpress).

### Cell transfection and interference

The coding sequences of *ACSM2A* (mRNA ID: NM_001010845) were amplified from HK-2 cells complementary DNAs. Then, the CDS of *ACSM2A* was cloned into a pcDNA 3.1 vector. Plasmids were transiently transfected into HK-2 cells with Lipofectamine 3000 Reagent (ThermoFisher) according to the manufacturer’s instructions.

To silence the *ACSM2A* gene, HK-2 cells were transfected with Human *ACSM2A* siRNA and *ZBP1* siRNA respectively. For control cells, HK-2 cells were transfected with control siRNA (control siRNA; Cat. No. Sc-37007; Santa Cruz). Cells were plated in plates at 50–60% confluency and transfected with RNAiMAX (Thermo Fisher, Invitrogen) transfection reagent and 50 nM siRNA.

The following siRNA sequences were employed:

SiRNA human *ACSM2A* forward: CCA​GUU​AUC​CAA​UCA​AGA​GUA​TT

SiRNA human *ACSM2A* reverse: UAC​UCU​UGA​UUG​GAU​AAC​UGG​TT

SiRNA human *ZBP1* forward: GGA​UGA​GCA​GUC​CAA​AGC​ATT

SiRNA human *ZBP1* reverse: UGC​UUU​GGA​CUG​CUC​AUC​CTT

### RNA extraction and quantitative real-time RT-PCR

Total RNA was extracted from the TECs or HK-2 cells using TRIzol reagent (Thermo Fisher, Invitrogen, 15596026CN), and the RNA was reverse transcribed to synthesize cDNA using HiScript III All-in-one RT SuperMix Perfect for qPCR (Vazyme, R333-01) following the manufacturer’s instructions. Quantitative real-time PCR (qRT-PCR) was conducted using ChamQ Universal SYBR qPCR Master Mix (Vazyme, Q711) on a LightCycler-480 Instrument (Roche). The relative mRNA quantities are expressed as 2^−ΔΔCT^.

The following qRT-PCR primers were employed:

Mouse *Acsm2* forward, 5′-ACT​AAT​ACC​CAT​ACA​GTG​GGG​C-3′,

Mouse *Acsm2* reverse, 5′-CTG​AAG​ATC​TCT​TGC​CAG​CCT​TC-3’;

Mouse *Gapdh* forward, 5′-GGT​GAA​GGT​CGG​TGT​GAA​CG-3′,

Mouse *Gapdh* reverse, 5′-CTC​GCT​CCT​GGA​AGA​TGG​TG-3’;

Human *ACSM2A* forward, 5′-CAG​AAG​GAG​ACA​TTG​GCA​TCA​GG-3′,

Human *ACSM2A* forward, 5′-AGT​CTC​CTC​GAA​TGT​TGG​CTG​C-3’;

Human *GAPDH* forward, 5′-GTC​TCC​TCT​GAC​TTC​AAC​AGC​G-3′,

Human *GAPDH* reverse, 5′-ACC​ACC​CTG​TTG​CTG​TAG​CCA​A-3’.

### Immunoblot analysis

Total proteins were isolated from the TECs or HK-2 cells with RIPA buffer (Beyotime). Total protein was quantified using the BCA protein assay kit (Thermo Scientific). Proteins were electrophoresed by SDS-PAGE, transferred to PVDF membranes, blocked with 5% skim milk (60 min, room temperature) and incubated with primary antibodies overnight at 4 °C: anti-Acsm2/ACSM2A (1:1000; abcam, ab181865), anti-GAPDH (1:10000; proteintech, 60004-1-Ig), anti-Caspase 3 (1:1000, CST, # 9662), anti-cleaved caspase 3 (1:1000; CST, #9661), anti-MLKL (1:5000; proteintech, 66675-1-Ig), anti-Phospho-MLKL (1:1000; CST, #91689), anti-Gasdermin D (1:1000, CST, # 39754), anti-Cleaved Gasdermin D (1:1000; CST, # 36425). After being washed with PBST, the PVDF membranes were incubated with anti-rabbit/mouse IgG, HRP-linked Antibody (1:2000; CST, #7074/#7076) at room temperature for 1 h and then detected with ECL (Thermo Scientific).

### LDH assay

LDH release was detected as an indicator of cytotoxicity using the LDH Assay Kit (Cytotoxicity) (Abcam, ab65393), according to the manufacturer’s instructions. The LDH released into the culture medium of cells was presented as a percentage of the total LDH.

### Real-time cell death analysis

Real-time cell death analysis was performed as previously described (Wang et al., 2025). Real-time cell death assays were performed using an IncuCyte S3 imaging system (Sartorius). HK-2 cells were seeded in 96-well plates. After indicated treatment, 100 nM SYTOX Green (Thermo Fisher Scientific, S7020) was added. The images were acquired at 37 °C and 5%CO2. The resulting images were analysed using the software package supplied with the IncuCyte imager, which counts the number of Sytox Green-positive HK-2 nuclei (Sytox + HK-2 nuclei) present in each image.

### ATP measurements assay

Cells ATP measurements were obtained using Enhanced ATP Assay Kit (Beyotime, S0026) following the manufacturer’s instructions. After centrifugation, cell debris was removed, and the supernatant was added into the substrate solution. The luminescence of solution was recorded in an Illuminometer with an integration time of 1 s per well. The protein content was quantified using the BCA protein assay kit (Thermo Scientific), and then the ATP concentration was converted to nanomoles per milligram of protein

### Mitochondrial or cellular ROS detection

Mitochondrial or cellular ROS levels were assessed using the MitoSOX Red mitochondrial superoxide indicator (HY-D1055; MedChemExpress) or the DCFH-DA (HY-D0940; MedChemExpress) according to the manufacturer’s instructions. Briefly, HK-2 cells were seeded in 96-well plates and subjected to the indicated treatments. Cells were then stained with 1 μM MitoSOX Red or 10 μM DCFH-DA for 30 min at room temperature to detect mitochondrial or cellular ROS. After washing twice with PBS, fluorescence was detected using an IncuCyte S3 imaging system (Sartorius), and fluorescence intensity was quantified with IncuCyte software.

### Immunofluorescence

Cells were grown on coverslips and treated as described. After washing in PBS, cells were fixed with 4% PFA for 20 min, permeabilized with 0.5% Triton X-100 in PBS for 15 min, blocked with blocking buffer (5% Skimmed milk diluted in PBS) for 1 h at RT, stained with primary antibodies overnight at 4 °C, and stained with secondary antibodies for 1 h at RT. Then, coverslips were mounted with DAPI-Fluoromount-G reagent (SouthernBiotech, 0100–20) for imaging. The following primary antibodies were used: Anti-Z-DNA/Z-RNA (Z22) (Absolute, Ab00783-23.0, 1:200 for IF). Secondary antibodies: Alexa488 (Thermo, A32731, 1:1000 for IF).

### Statistical analysis

All experiments were conducted in at least three independent biological replicates, and results are presented as the mean ± standard deviation (SD). Technical replicates were used only to ensure measurement consistency and were averaged prior to statistical analysis. For statistical evaluation, a normal distribution of the data was assumed, and analyses were carried out using GraphPad Prism version 11 (GraphPad Software, United States of America). Comparisons between two groups were performed using an unpaired, two-tailed Student’s t-test. For experiments involving more than two groups with a single independent variable, one-way analysis of variance (ANOVA) followed by Tukey’s *post hoc* test was applied. A p value of less than 0.05 was regarded as statistically significant.

## Data Availability

The original contributions presented in the study are included in the article/Supplementary Material, further inquiries can be directed to the corresponding author.
